# Perceptions of Long-Acting Injectable Pre-Exposure Prophylaxis Among Men Who Have Sex With Men and Transgender Individuals in Europe Using Structural Text Modeling Technique: Cross-Sectional Study

**DOI:** 10.2196/72491

**Published:** 2025-09-12

**Authors:** Haoyi Wang, Johann Kolstee, Julio Croce Martinez, David van de Vijver, Jonathan Tosh, Melanie Schroeder, Ama Appiah, Hanne ML Zimmermann, Kai J Jonas

**Affiliations:** 1 Department of Work and Social Psychology Maastricht University Maastricht The Netherlands; 2 Department of Viroscience Erasmus MC Rotterdam The Netherlands; 3 ViiV Healthcare Ltd London United Kingdom

**Keywords:** men who have sex with men, trans*, pre-exposure prophylaxis, long-acting injectable PrEP, qualitative, topic modeling, Europe

## Abstract

**Background:**

Oral pre-exposure prophylaxis (PrEP) effectively prevents HIV but remains unevenly accessible across Europe. Long-acting PrEP (LA-PrEP), recently approved in Europe, offers new HIV protection options. However, no qualitative evidence is available to inform people’s perceptions of this novel modality.

**Objective:**

This study provides the first large-scale, qualitative evidence in English from 20 European countries on how men who have sex with men (MSM) and trans* individuals perceive LA-PrEP.

**Methods:**

We analyzed open-ended responses from 3123 HIV-negative MSM and trans* individuals from 20 European countries who completed the PROTECT survey in English. Participants were asked to describe what LA-PrEP means to them using words or short phrases. We used word clouds for initial insights and structural topic modeling to identify topics and explore their relevance across socioeconomic status, migration background, oral PrEP use, and affordability and the association of these perceptions with LA-PrEP intention.

**Results:**

The responses reflected generally positive associations toward LA-PrEP, with the most frequently mentioned word being “safe,” “freedom,” and “convenient.” However, some ambivalent and negative perceptions, such as “nothing,” “unknown,” “dunno,” and “unnecessary,” were also noted. Structural topic modeling identified 5 main response topics: safety, empowerment, convenience/reliability, peace of mind, and concerns/uncertainties. The empowerment offered by LA-PrEP was the most prominent topic, representing one third (28.1%) of the responses, followed by safety (21%), convenience and reliability (16%), and peace of mind (15%), while concerns/uncertainties made up 20%. Variation in the relevance of these topics was found, showing LA-PrEP being seen as more empowering (=.070, 95% CI 0.042-0.097) and convenient (=.057, 95% CI 0.034-0.081) by current oral PrEP users, but less empowering (=–.052, 95% CI –0.087 to 0.017) and convenient (=–.034, 95% CI –0.064 to 0.005) for individuals in countries with limited oral PrEP access and affordability. The topic of safety was more relevant among those with lower levels of education (=.052, 95% CI 0.022-0.083) and those living in a country where PrEP was not reimbursed (=.035, 95% CI 0.002-0.069), but less relevant among current oral PrEP users (=–.094, 95% CI –0.12 to 0.066). We also found that ambivalent and negative perceptions were less relevant among current oral PrEP users (=–.032, 95% CI –0.056 to 0.007) and were negatively associated with a lower intention to use LA-PrEP (=–.075, 95% CI –0.101 to 0.005).

**Conclusions:**

Our research showed that MSM and trans* individuals in Europe generally have a positive outlook on LA-PrEP, suggesting it is likely to be well accepted upon its introduction in Europe. However, a subset of the target population may be hesitant to adopt LA-PrEP, underscoring the need for alternative HIV prevention strategies tailored to these individuals. To support potential future LA-PrEP implementation in Europe and to maximize its impact, appropriate communication strategies are essential for supporting informed decision-making.

## Introduction

HIV oral pre-exposure prophylaxis (PrEP) has proven to be highly effective in preventing HIV [1,2] and is widely accepted by key populations affected by HIV [3,4]. By 2024, approximately 3.5 million individuals worldwide were using PrEP [5]. Despite efforts to increase its availability and accessibility, PrEP uptake remains lower than expected in many countries and among key populations [6-8], falling short of the United Nations 2025 target of reaching 10 million users globally. The full potential of oral PrEP at the population level has yet to be reached [9-12].

Recently, novel long-acting (LA) PrEP formulations have emerged to address the limitations of oral PrEP [13-17] and enhance PrEP coverage and its impact at the population level [18-21]. Notably, cabotegravir administered every 2 months has demonstrated statistically superior efficacy in preventing HIV compared to oral PrEP across all key populations [22,23]. Recognizing this, the World Health Organization (WHO) endorsed cabotegravir for high-risk groups [24]. Cabotegravir was subsequently approved for use in the United States in 2022 and in Europe in 2023 [25,26]. In addition, the newer LA injectable lenacapavir, administered twice yearly, has recently demonstrated 100% efficacy in preventing HIV among cisgender women [27].

Understanding the views and perceptions of novel LA-PrEP among potential end users is crucial, particularly in Europe, where data on LA-PrEP perceptions and attitudes are scarce [28]. As of early 2025, only one systematic review has synthesized global perspectives on LA-PrEP among key populations [16], reporting that while LA-PrEP is generally perceived as effective, easy to use, and convenient, concerns remain about potential pain, side effects, and logistical challenges such as the need for regular appointments. However, these insights stemmed primarily from qualitative studies with small sample sizes, raising questions about their generalizability. Furthermore, most of the available evidence originates from North America and Africa, with only 5 studies focusing on Europe, none of which provided qualitative data on European key populations’ expectations, attitudes, or perceptions regarding LA-PrEP, and none included trans* participants. This gap underscores the need for large-scale qualitative evidence specific to the European context to inform the introduction and scaling of LA-PrEP.

Previous research has highlighted that perceptions and preferences for LA-PrEP vary significantly among subgroups within key populations [16]. Drawing from experiences with oral PrEP in Europe, individuals with lower socioeconomic positions (SEP) and migration backgrounds were less likely to access oral PrEP, a pattern that may similarly influence perceptions and access to LA-PrEP [4,7,29,30]. In addition, past and current experiences with oral PrEP can shape perceptions of LA-PrEP [31]. For example, PrEP-naïve MSM in Europe are less likely to intend to use LA-PrEP compared to current users [28], indicating underlying differences in attitudes and expectations. The LA nature of LA-PrEP, which alleviates the burden of daily pill-taking, may particularly appeal to individuals who experience challenges with adherence to daily regimens [19,22]. However, beyond individual-level considerations, structural factors such as affordability and health system infrastructure are also critical. In countries where oral PrEP is not yet reimbursed or remains unaffordable, LA-PrEP may be viewed differently than in countries with more accessible PrEP options [7,32]. Additional structural barriers—including provider bias, mismatches in provider-client expectations [33], and geographic distance to PrEP clinics [34,35]—may further shape individuals’ LA-PrEP intention and uptake. Understanding these subgroup variations emphasizes the importance of analyzing large-scale qualitative data to gain insights into how different populations perceive LA-PrEP and how these perceptions may influence their intention to use it. Such insights are essential for informing European access policies and guidelines to ensure that the introduction and scale-up of LA-PrEP meet the diverse needs of key populations.

Gathering large-scale qualitative data is, however, often challenging and costly, leading to studies with smaller sample sizes [36]. One potential solution is incorporating open-ended responses into large-scale quantitative cross-sectional surveys. Yet, most global HIV prevention surveys, including those on PrEP [24,37-39], rely on closed-ended questions, which limit responses to predefined options and scales. To address these limitations, text mining and topic modeling techniques have emerged as scalable methods to analyze qualitative responses. However, their application in HIV prevention research, particularly for understanding end-user perceptions, remains limited. Most existing work is typically applied to public discourse, such as user reactions on social media platforms such as X (formerly Twitter) or Instagram. While such studies offer insights into general sentiment or advocacy trends, they are constrained by the absence of detailed respondent information, making subgroup analysis difficult. Moreover, topics tend to reflect political or public health narratives rather than individual perspectives or lived experience. Only a few studies have applied text mining and topic modeling to HIV-related content. For instance, BERTopic has been used to examine trends in HIV care services literature [40], and topic modeling has been used to explore public discussions of PrEP on social media [41-43], demonstrating the potential of such tools to synthesize broad themes. However, none of these approaches has been used to analyze individual-level qualitative data on LA-PrEP, particularly within a European context.

Among topic modeling methods, structural topic modeling (STM) is uniquely suited to social science and public health research [44,45]. Unlike other unsupervised methods such as keyword-assisted topic modeling [46], seeded latent Dirichlet allocation [47], or BERTopic [48], STM uniquely allows the inclusion of metadata, such as socioeconomic status or previous PrEP experience, as covariates that influence topical prevalence and content [45,49,50]. This makes STM especially valuable for hypothesis-driven or exploratory analysis, enabling the exploration of how themes or topics vary across different subgroups—something rarely possible in social media–based or literature-based studies, and ideal for analyzing open-ended survey responses in social science and public health domains [44]. By integrating STM with large-scale, individual-level qualitative data, we offer a novel methodological approach to understand how key populations perceive LA-PrEP, filling an important gap in both computational social science and HIV prevention research.

Taken together, this study aimed to provide the first large-scale qualitative insight into the attitudes and perceptions of LA-PrEP among MSM and trans* individuals in Europe. In addition, we sought to understand whether these perceptions differ by subgroup characteristics and previous oral PrEP use, and whether they are associated with intention to use of LA-PrEP using an STM approach.

## Methods

### Study Recruitment and Study Population

We conducted an internet-based survey (PROTECT study) across 20 European countries (Austria, Belgium, Cyprus, the Czech Republic, Denmark, Finland, France, Germany, Greece, Ireland, Italy, Luxembourg, the Netherlands, Norway, Poland, Portugal, Spain, Sweden, Switzerland, and the United Kingdom) among MSM and trans* individuals. The PROTECT survey aimed to better understand the characteristics and behavior of key populations in Europe who may benefit from new PrEP modalities. Participants were recruited using a convenience sampling approach. Ads containing text information about the purpose of the study, photo images, and inbox messages were used to solicit potential participants mainly from gay dating apps (eg, Grindr) and through a social media campaign on Instagram and Facebook during a period of 6 months (October 2023-April 2024). Participants could fill in the survey in 22 different languages. Overall, 63.4% of the participants who started the survey completed at least 95% of it, resulting in 15,428 final survey responses. For this study, we included only HIV-negative participants who responded to the survey in English. The full study procedure, the full survey, and the recruitment approach have been described elsewhere [51-53].

### Measures

In the survey, participants were asked to describe what LA-PrEP meant to them using words or short phrases. To analyze European MSM and trans* individuals’ perceptions of LA-PrEP, we first processed their responses by normalizing the open response entries—removing punctuation and numbers, converting all words to lowercase, and removing stop words [54]. Since most responses were single words, additional text preparation was required for robust model performance [49,55,56]. In this text normalization preparation process, to reduce dimensionality and improve coherence, we manually standardized entries, aligning them with the most frequently entered term to avoid having 2 or more expressions of the same word stem [57]. For instance, if “safe” appeared more often than “safety,” we replaced all instances of “safety” with “safe” to maintain consistency and reflect the most common expressions used by participants. In addition, for short phrases that indicated the same meaning as a single word, we manually replaced these short phrases with the existing single words to ensure key information would not be removed during text cleaning [58]. For example, we replaced “I don’t know” with “dunno.” Finally, we used abbreviations for multiple-word phrases that would otherwise lose their meaning, such as converting “peace of mind” to “pom” [59]. This text normalization step was conducted by HW and KJJ to ensure consistency, with intercoder agreement established on all final decisions. The raw data and entries after manual cleaning are shown in Multimedia Appendix 2.

To explore potential variations in different meanings and expectations of LA-PrEP between groups, we also measured participants’ SEP, migration background, oral PrEP use history, PrEP affordability, and LA-PrEP intention. SEP variables included perceived income, education, and migration background. Oral PrEP use variables included uptake, regimens, and adherence. PrEP affordability was proxied by the current reimbursement policies of the participant’s country of residence (Table 1). We considered a country to have the fully-reimbursed status if PrEP was being included in health insurance coverage (Denmark, Finland, France, Ireland, Luxembourg, Norway, Portugal, Spain and the United Kingdom), the partially-reimbursed status if a copayment was needed for PrEP access (Austria, Belgium, Germany, Netherlands, Sweden and Switzerland), and the non-reimbursed status if PrEP was only accessible via out-of-pocket payment (Cyprus, Czech Republic, Greece, Italy, and Poland). The classification of countries was based on policy settings up to and including January 1, 2023. LA-PrEP intention was dichotomized as “higher intention” and lower intention.” Variable descriptions can be found in Table 2.

**Table 1:** Country classification of pre-exposure prophylaxis (PrEP) reimbursement status as of January 1, 2023, which was relevant for the sampling period of October 2023-April 2024.

PrEP reimbursement status	Countries
Fully reimbursed	Denmark, Finland, France, Ireland, Luxembourg, Norway, Portugal, Spain, and the United Kingdom
Partially reimbursed	Austria, Belgium, Germany, the Netherlands, Sweden, and Switzerland
Nonreimbursed	Cyprus, the Czech Republic, Greece, Italy, and Poland

**Table 2:** Sample characteristics of 3123 HIV-negative men who have sex with men and trans* individuals who completed the PROTECT survey in English across 20 European countries from October 2023-April 2024.

Characteristic			Value, n (%)
**Age (years)**			
	18-24	273 (8.7)	
	25-29	462 (14.8)	
	30-39	1037 (33.2)	
	40-49	702 (22.5)	
	50-59	374 (12)	
	60-69	161 (5.2)	
	70+	114 (3.7)	
**Education**			
	Below Bachelor’s degree	705 (22.6)	
	Bachelor’s or above	2418 (77.4)	
**Perceived income**			
	Lower	1811 (58)	
	Higher	1312 (42)	
**Migration status**			
	Nonmigrant	1467 (47)	
	Migrant	1656 (53)	
**Oral PrEP use**			
	Current users	1710 (54.8)	
	Noncurrent users	1413 (45.2)	
**Oral PrEP^a^ regimen^b^**			
	Daily	971 (56.8)	
	Event-driven	487 (28.5)	
	Mixed-use	252 (14.7)	
**Oral PrEP adherence^c^**			
	Optimal	1056 (72.4)	
	Suboptimal	402 (27.6)	
**PrEP affordability**			
	Fully reimbursed	1758 (56.3)	
	Partially reimbursed	593 (19)	
	Nonreimbursed	772 (24.7)	
**Long-acting PrEP intention**			
	Yes	2306 (73.8)	
	No	817 (26.2)	

^a^PrEP: pre-exposure prophylaxis.

^b^Available only to participants currently using oral PrEP.

^c^Available only to those who reported currently using daily or event-driven PrEP.

### Statistical Analysis

#### Descriptive Analysis

We performed descriptive statistical analyses on all independent variables. For participants’ perceptions of LA-PrEP, we generated word clouds of the 50 most frequently mentioned terms, both for the overall sample and within subgroups based on SEP, migration background, oral PrEP use, PrEP affordability, and LA-PrEP intention, to provide a visual overview.

#### Structural Topic Modeling

In our STM analysis, the number of topics was not prespecified but instead was determined by statistical evaluation of models with varying numbers of topics, ranging from 3 to 10. After text normalization and data cleaning (described previously in the “Measures” section), we assessed the statistical fit of each K-topic model by examining several diagnostic metrics: held-out likelihood, which reflects the (log-)likelihood of estimating the probability of unseen documents given a trained model and thus indicates predictive performance [60]; residual analysis, which evaluates how well the model captures word co-occurrence patterns by checking for remaining correlation between words after accounting for topics [61]; semantic coherence, which measures the degree to which words within a topic co-occur, reflecting topic interpretability [62]; and the lower bound, which serves as an indicator of model convergence during variational inference [62]. We considered models with higher held-out likelihood, lower residuals, and greater semantic coherence to be better fitting, and those with higher lower bound values to exhibit more robust convergence. Based on these criteria, we selected the model that demonstrated the best balance of performance and convergence across all metrics.

We then manually explored the optimal model with the best fit, evaluating the relevance of each topic in terms of the meanings and expectations of LA-PrEP among MSM and trans* individuals. This included examining the top 6 highest probability words for each topic in a thematic approach, based on frequency and exclusivity (FREX) word profiling—FREX words are weighted based on their overall FREX to a topic [45]. For the sensitivity analysis, we evaluated each topic in three other word profiles, including the highest probability words, lift words, and score words [45]. We also estimated the topic relevance, which refers to the relative importance of these topics in the overall corpus [63], by assessing the proportion of a document devoted to a certain topic [64].

Finally, to explore variations in the relevance of LA-PrEP meanings and expectations across different subgroups of MSM and trans* individuals, we conducted univariable linear topic regressions. These regressions used the topic proportion of each estimated topic as the dependent variable, considering factors such as SEP, migration background, oral PrEP use, PrEP affordability, and LA-PrEP intention based on the text corpus. Such regressions allow us to assess the conditional expectation of topic prevalence given document characteristics [65]. We conducted only univariable topic regression to explore how individual covariates relate to topic prevalence, rather than using a multivariable STM model. Given the exploratory nature of our analysis, this approach allowed us to examine the influence of each covariate independently without introducing potential confounding or multicollinearity. Several covariates in our dataset were correlated (eg, education, income, and migration status or oral PrEP use, adherence, and regimens), and including them simultaneously in a multivariable model could lead to unstable estimates and reduced interpretability. Moreover, univariable models help to avoid overfitting and ensure more stable estimation, especially in the presence of small subgroups and a relatively large number of topics. This strategy provided a clear first step in identifying patterns of interest for future hypothesis-driven analysis. All analyses were performed using R Foundation for Statistical Computing (version 4.3.2).

### Ethical Considerations

This study is reported following the STROBE (Strengthening the Reporting of Observational Studies in Epidemiology) guidelines for reporting observational studies (Multimedia Appendix 5) [66]. Ethical approval was obtained from the Ethics Review Committee Psychology and Neuroscience at Maastricht University (OZL_262_08_01_2023_S21). All PROTECT participants provided written informed consent at the time of enrollment. The data collected were fully anonymized and cannot be traced back to individual participants. No financial or other form of compensation was provided to participants in the PROTECT study.

## Results

### Sample Characteristics

In this study, 3123 HIV-negative MSM and trans* individuals completed the PROTECT survey in English and were included, with a median age of 37 (IQR 30-46) years. Of these participants, most had an educational attainment level of a bachelor’s degree or above (n=2418, 77%), more than half perceived a lower income (n=1811, 58%), and more than half reported having a migration background (n=1656, 53%). Among those who reported a migration background, the majority (n=2500, 62%; Table S1 in Multimedia Appendix 1) were living in non-English–speaking countries such as Germany (n=214, 12.9%) and the Netherlands (n=194, 11.7%), while 38% (n=623) were living in English-speaking countries, including Ireland (n=147, 8.9%) and the United Kingdom (n=476, 28.7%).

Overall, more than half of the participants (n=1710, 55%) were currently using oral PrEP, with the majority (n=971, 57%) reporting daily PrEP use, followed by event-driven PrEP (n=487, 28%) and 15% (n=252) reporting a mix of daily and event-driven PrEP use. Notably, a significant proportion of those using daily and event-driven PrEP reported suboptimal adherence (n=402, 28%). Regarding PrEP affordability, the majority lived in countries where oral PrEP was fully reimbursed (n=1758, 56%), while 19% (n=n=772) lived in countries where oral PrEP was not reimbursed. In addition, most participants (n=2306, 74%) indicated their intention to use LA-PrEP if it became available and affordable. Detailed sample characteristics are provided in Table 2.

### Word Associations With LA-PrEP

The overall word cloud highlighting the 50 most frequently mentioned words associated with LA-PrEP is shown in Figure S2 in Multimedia Appendix 1. Descriptively, the responses indicated generally positive associations with LA-PrEP. The most frequently mentioned words were “safe,” followed by “freedom,” and “convenient.” However, ambivalent and relatively negative perceptions were also noted, including terms such as “nothing,” “unknown,” “dunno,” and “unnecessary,” as well as perceived side effects such as “pain.”

Variations in perceptions of LA-PrEP were observed across different SEP subgroups. Lower-income participants frequently used terms such as “safe” and more ambivalent phrases such as “nothing” or “unknown,” while higher-income participants emphasized “convenient” (Figure S1 in Multimedia Appendix 1). Education also showed notable differences: those with a bachelor’s degree or higher often mentioned “freedom” and “convenient,” whereas individuals with lower education mostly used “safe” and fewer terms overall (Figure S2c and S2d in Multimedia Appendix 1). Similar patterns emerged between participants with and without a migration background (Figure S1 in Multimedia Appendix 1).

Among oral PrEP use subgroups, current users highlighted LA-PrEP as “convenient” and associated it with “freedom,” whereas nonusers focused on “safe” and used more ambivalent terms such as “nothing” and “dunno” (Figure S3A and S3B in Multimedia Appendix 1). Daily PrEP users anticipated greater convenience and expressed fewer negative views compared with nondaily users (Figure S2C and S2D in Multimedia Appendix 1). Furthermore, users with suboptimal adherence expected more “freedom” from LA-PrEP and had fewer negative perceptions than those with optimal adherence (Figure S2e and S2f in Multimedia Appendix 1).

Variations were also noted regarding PrEP affordability and intentions to use LA-PrEP. Participants from countries with full PrEP reimbursement had more positive views and frequently used “freedom,” while those from countries with partial or no reimbursement mentioned it less (Figure S4A-C in Multimedia Appendix 1). A significant difference was observed between participants with high and low intentions to use LA-PrEP. Those with high intentions expressed almost exclusively positive views, while those with low intentions frequently used ambivalent or negative terms such as “nothing,” “unknown,” “uncertainty,” and “unnecessary” (Figure S4D and S4E in Multimedia Appendix 1). Detailed word frequencies are shown in Multimedia Appendix 3.

### Structural Topic Modeling

#### Model Selection

The model with 5 topics was selected based on a comprehensive evaluation of multiple diagnostic metrics, which collectively indicated an optimal balance between model fit, interpretability, and generalizability (Figure S5 in Multimedia Appendix 1). In terms of held-out likelihood, a measure of predictive performance, the top-performing models were those with 3, 5, and 10 topics. Although the models with 3 and 10 topics showed slightly higher held-out likelihoods, the model with 10 topics also had the highest residuals, suggesting potential overfitting and a poorer capture of word co-occurrence patterns. This could introduce greater complexity and increase the risk of thematic redundancy. Conversely, the model with 3 topics, while simpler, had the lowest semantic coherence and weaker convergence based on lower bound values. In contrast, the model with 5 topics offered the best overall trade-off, demonstrating improved held-out likelihood, higher semantic coherence, lower residuals, and robust model convergence. Therefore, it was selected as the most balanced and interpretable solution.

#### Topic Interpretation

Table 3 presents the FREX word profile, interpretation, and relevance of each topic of the meanings and expectations of LA-PrEP. Topic 1 (safety) captures the expectation that LA-PrEP will be a safe option with long-lasting protection against HIV. Topic 2 (empowerment) emphasizes the perception that LA-PrEP is a suitable method of HIV prevention that increases the sense of agency among future users. Topic 3 (peace of mind [POM]) highlights that LA-PrEP is an innovative option that is practical to use and provides a feeling of calmness and nonagitation. Topic 4 (convenience and reliability) focused on confidence in the effectiveness of LA-PrEP. Topic 5 (concerns and uncertainties), conversely, reflected ambivalent and relatively negative perceptions of LA-PrEP. No major differences were found between different word profiling methods (Table S2 in Multimedia Appendix 1).

Other word profiles based on other methods can be found in Table S1.

Based on our document-topic proportion estimations (Table 2; Figure S6 in Multimedia Appendix 1), the topic of empowerment emerged as the most relevant, accounting for 28% of the associations with and expectations of LA-PrEP. This suggests that nearly one third of MSM and trans* participants anticipated that LA-PrEP would be an empowering HIV prevention option. The second most prominent topic was safety (21%), reflecting the importance placed on the superior safety profile of LA-PrEP. Following this were the topics of concerns and uncertainties (20%), convenience and reliability (16%), and POM (15%).

**Table 3:** Topic word profiles, relevance, and interpretations of the open responses to the question, “What does LA-PrEP mean to you?” from 3123 HIV-negative men who have sex with men and trans* individuals who completed the PROTECT survey in English across 20 European countries from October 2023-April 2024.

Topic	Relevance	FREX^a^ word profile	Interpretation	Interpretation keyword
Topic 1	20.7%	Long, safe, last, protect, access, and worry	LA-PrEP^b^ will be a safe option that has long-lasting protection	Safety
Topic 2	28.1%	Freedom, easy, interest, alternative, pain, and better	Even though LA-PrEP may lead to some pain, it is an alternative that can be interesting, easy, and empowering for more freedom	Empowerment
Topic 3	15%	Reassurance, secure, practical, simple, innovative, and use	LA-PrEP as an innovative option may bring POM^c^ by being practical	POM
Topic 4	16.2%	Convenient, prevent, reliable, hope, inject, and HIV	LA-PrEP is an injectable PrEP^d^ for HIV that can be convenient and reliable	Convenient and reliable
Topic 5	20%	Nothing, option, unknown, NA^e^, unnecessary, and dunno	Concerns and uncertainties on LA-PrEP	Concerns and uncertainties

^a^FREX: frequency and exclusivity.

^b^LA-PrEP: long-acting pre-exposure prophylaxis.

^c^POM: peace of mind.

^d^PrEP: pre-exposure prophylaxis.

^e^NA: not available.

#### Topic Regressions

Multimedia Appendix 4 summarizes the topic regressions between each estimated topic and SEP, migration background, oral PrEP use, PrEP affordability, and LA-PrEP intention subgroups. For the topic of safety, compared to those with a bachelor’s degree or higher, participants with education below a bachelor’s degree were significantly more likely to discuss this theme, with a 5.2-percentage point higher expected topic probability (=.052, 95% CI 0.022-0.083). Safety concerns were also 3.5 percentage points more prevalent among participants living in countries where PrEP was not reimbursed (=.035, 95% CI 0.002-0.069). In contrast, current oral PrEP users were 9.4 percentage points less likely to discuss safety as a concern (=–.094, 95% CI –0.123 to 0.066). The topic of empowerment was significantly more prevalent among current oral PrEP users, who showed a 7.0 percentage point higher expected topic probability compared to nonusers (=.070, 95% CI 0.042-0.097), and among those with a higher intention to use LA-PrEP (5.1 percentage point increase; =.051, 95% CI 0.017-0.085). In contrast, it was 5.2 percentage points less prevalent among participants living in countries where PrEP was not reimbursed (=–.052, 95% CI –0.087 to 0.017). The topic of LA-PrEP being convenient or reliable showed a similar pattern. It was 5.7 percentage points more prevalent among current oral PrEP users (=.057, 95% CI 0.034-0.081) and 3.2 percentage points more prevalent among those with a higher intention to use LA-PrEP (=.032, 95% CI 0.003-0.062). However, it was 3.7 percentage points less prevalent among individuals currently using event-driven oral PrEP (=–.037, 95% CI –0.078 to 0.003) and 3.4 percentage points less prevalent among participants in countries where PrEP was nonreimbursed (=–.034, 95% CI –0.064 to –0.005). For the topic of concerns or uncertainties, prevalence was 3.2 percentage points lower among current oral PrEP users (=–.032, 95% CI –0.056 to –0.007) and 7.5 percentage points lower among those with higher intention to use LA-PrEP (=–.075, 95% CI –0.101 to –0.005). For the topic of POM, we did not find any significant differences across the subgroups.

## Discussion

### Principal Findings

To our knowledge, this is the first study to explore views and perceptions of LA-PrEP in Europe and the first to apply STM to large-scale qualitative data from open-ended survey responses on LA-PrEP perceptions. Our findings demonstrate that machine learning methods can effectively analyze large-scale qualitative data in HIV prevention, enhancing the capabilities of human coders. These techniques can be applied to other topics where qualitative evidence is needed. Consistent with a previous systematic review [16], this study found generally positive views of LA-PrEP among MSM and trans* individuals across Europe, suggesting that it is likely to be well received alongside existing oral PrEP options once it becomes available. However, the study also uncovered significant concerns and uncertainties, accounting for 20% of topic relevance, regarding LA-PrEP among key populations. While this proportion reflects meaningful apprehension, it is somewhat lower than levels of concern typically reported in earlier qualitative research on novel biomedical interventions, such as initial PrEP rollout [67,68] or vaccine hesitancy studies [69,70]. Given that LA-PrEP is a new and potentially more invasive prevention method compared with oral regimens [71,72], ongoing attention to these concerns is warranted. We need to acknowledge that LA-PrEP may not be suitable for everyone, and alternative prevention options should remain accessible to maximize the public health impact of the current HIV prevention strategies.

Our models identified 5 main topics within these views and perceptions. Three of these were notably positive. These positive sentiments align with the intended benefits of LA-PrEP, which aim to address some of the challenges of oral PrEP [15,22]. Notably, the topics of empowerment and convenience and reliability were more relevant among current oral PrEP users, who also showed a higher intention to use LA-PrEP [28]. In contrast, these topics were less relevant to individuals in less privileged contexts, such as those in countries where oral PrEP is not or not fully reimbursed. Our findings thus underscore the importance of oral PrEP as a HIV prevention modality—without optimal oral PrEP services, LA-PrEP might not be viewed positively and might not effectively address the remaining unmet needs that key populations may experience. Further, the perception of LA-PrEP as convenient and reliable was less relevant for those using event-driven PrEP. Since this population typically experiences fewer challenges in using oral PrEP [73], LA-PrEP may not be as appealing to them as it is to other groups.

Conversely, the topic of the superior safety profile of LA-PrEP compared with oral PrEP was more relevant to individuals in less privileged circumstances, such as those with lower educational attainment or those living in countries where oral PrEP is not fully reimbursed. However, this topic was significantly less relevant to current oral PrEP users. One possible reason is that our study was conducted after the publication of cabotegravir efficacy data, which may be more accessible to those with greater privileges. Another reason could be the positive safety experiences reported by current oral PrEP users [74], suggesting that the safety profile of LA-PrEP may not be their primary concern.

Interestingly, concerns and uncertainties about LA-PrEP were significantly less relevant to current oral PrEP users, while remaining important for other subgroups. This aligns with previous findings that current oral PrEP users are more likely to adopt LA-PrEP compared with PrEP-naïve MSM [28,31]. Our qualitative evidence suggests that current users, familiar with and positive about PrEP [74], have fewer concerns about LA-PrEP as a new HIV prevention option, which may explain their higher intention to use it. Conversely, individuals with concerns or uncertainties are less likely to intend to use LA-PrEP. To facilitate the adoption of LA-PrEP, it is crucial for HIV prevention authorities, health care providers, and communities to address these concerns, especially among those who show interest. In addition, guidelines and support should be developed to help individuals navigate periods of use and nonuse or to switch between prevention regimens [6], ensuring they can make informed decisions.

### Limitations

Our study has some limitations. First, our analysis was based solely on an English-language corpus, which included responses from migrants from non-English–speaking countries who chose to complete the survey in English, as well as participants residing in the United Kingdom and Ireland. This choice was also driven by the lingua franca role of English in Europe. Translating non-English entries into English could potentially create translation or semantic bias and increase the risk of bias due to language-to-country matching in terms of determinants in the regression analysis. The fact that the regression results do not only exclusively reflect the situation in the United Kingdom and Ireland supports broader representation. To mitigate this risk, we prioritized semantic accuracy over broader representativeness. As a result, the generalizability and external validity of our findings may be limited, particularly among speakers of other mother tongues. Future research should explore perceptions of LA-PrEP in local languages to generate more region-specific and culturally specific insights, especially in settings where English is not the primary language. Second, our sample was recruited via an internet-based survey, likely resulting in an overrepresentation of digitally literate individuals with greater health engagement. This may limit the external validity of our findings, particularly for populations with limited internet access or digital literacy. Finally, we did not conduct in-depth interviews and relied solely on responses to open-ended survey questions. While STM is an innovative tool for analyzing large-scale qualitative data, it relies on probabilistic modeling, which may result in the loss of important contexts and meanings, introducing potential bias. Despite this, our findings are consistent with previous research outside Europe [16], supporting their ecological validity. Future work should complement these findings with qualitative interviews to provide deeper insight.

### Conclusions

Our research reveals a generally positive outlook on LA-PrEP, suggesting that it is likely to be well-accepted upon its introduction in Europe. However, notable concerns remain, indicating that a subset of the target population may be hesitant to adopt LA-PrEP, highlighting the need for alternative HIV prevention strategies tailored to these individuals. These concerns must be addressed among potential LA-PrEP users to ensure a successful implementation. To achieve broad coverage and maximize public health impact, the introduction of LA-PrEP must include evidence-based, tailored public health communication strategies that promote informed decision-making among diverse populations, thereby contributing to the global effort to end the HIV epidemic.

## Appendix

Multimedia Appendix 1 Additional figures and tables.

Multimedia Appendix 2 Raw data and entries after manual cleaning.

Multimedia Appendix 3 Detailed word frequencies.

Multimedia Appendix 4 Topic regression of the open responses of “What LA-PrEP means to you?” question from 3123 HIV-negative MSM and trans* individuals who completed the PROTECT survey in English across 20 European countries, October 2023-April 2024.

Multimedia Appendix 5 STROBE checklist.

## Abbreviations

FREX: frequency and exclusivity
LA: long-acting
POM: peace of mind
PrEP: pre-exposure prophylaxis
SEP: socioeconomic position
STM: structural topic modeling
STROBE: Strengthening the Reporting of Observational Studies in Epidemiology
WHO: World Health Organization

## Data Availability

The datasets generated or analyzed during this study are available from the corresponding author on reasonable request.
