# Supplementary material for: Perceptions of Long-Acting Injectable Pre-Exposure Prophylaxis Among Men Who Have Sex With Men and Transgender Individuals in Europe Using Structural Text Modeling Technique: Cross-Sectional Study
Source: JMIR Public Health Surveill. 2025 Sep 12;11:e72491. doi: 10.2196/72491 (PMC12475884; doi:10.2196/72491)
Supplement: Multimedia Appendix 1 [file publichealth_v11i1e72491_app1.docx]

# Multimedia Appendix 1

**Figure S1. The overall word cloud of the top 50 most frequently mentioned words of the meanings of LA-PrEP among MSM and trans* individuals.**

**
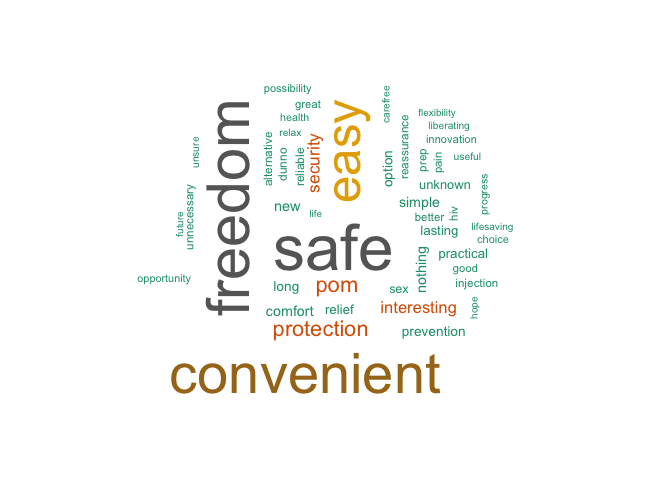
**

**Figure S2.** **The word clouds of the top 50 most frequently mentioned words of the meanings of LA-PrEP among MSM and trans* individuals across their socioeconomic positions.**


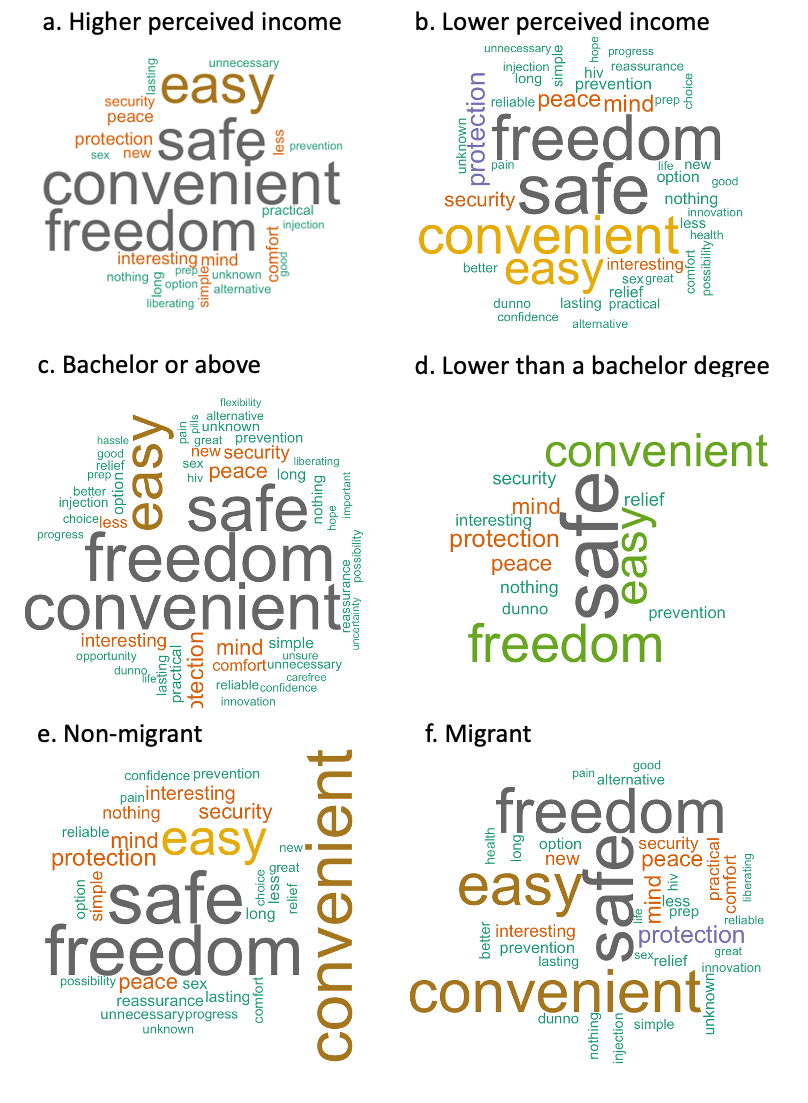


**Figure S3.** **The word clouds of the top 50 most frequently mentioned words of the meanings of LA-PrEP among MSM and trans* individuals across their oral PrEP use statuses.**


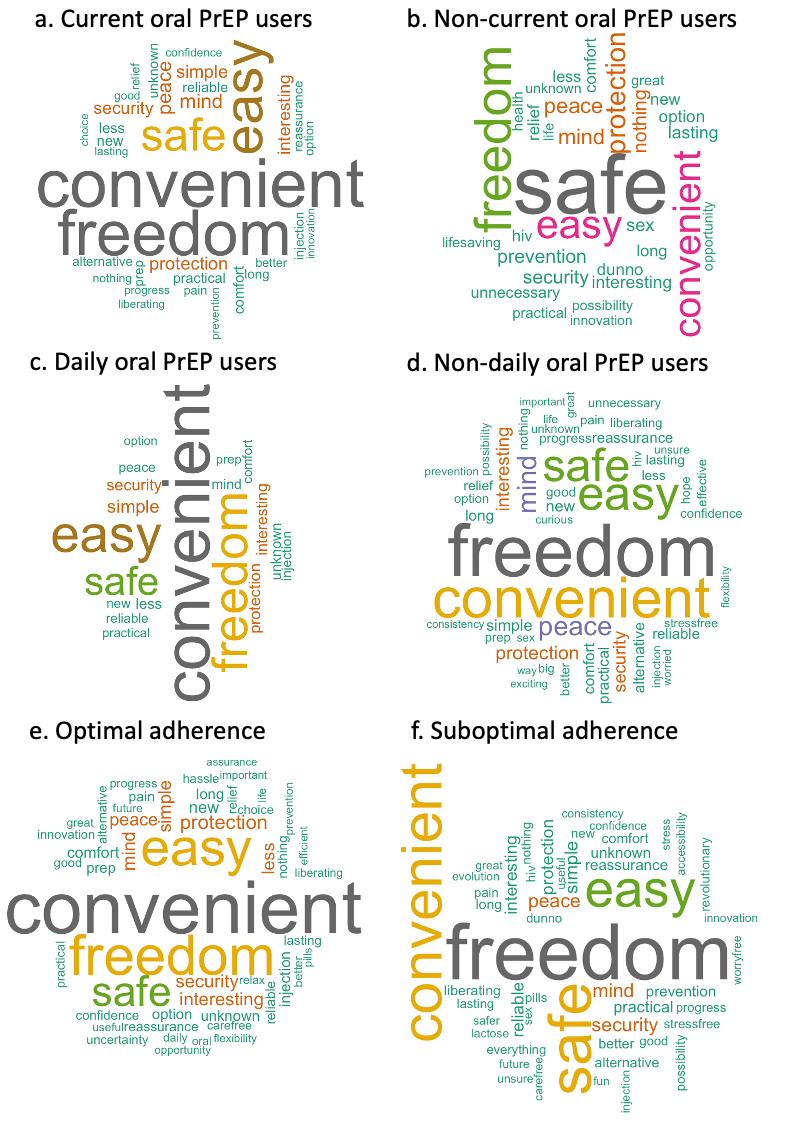


**Figure S4.** **The word clouds of the top 50 most frequently mentioned words of the meanings of LA-PrEP among MSM and trans* individuals across their PrEP affordability and LA-PrEP intention.**


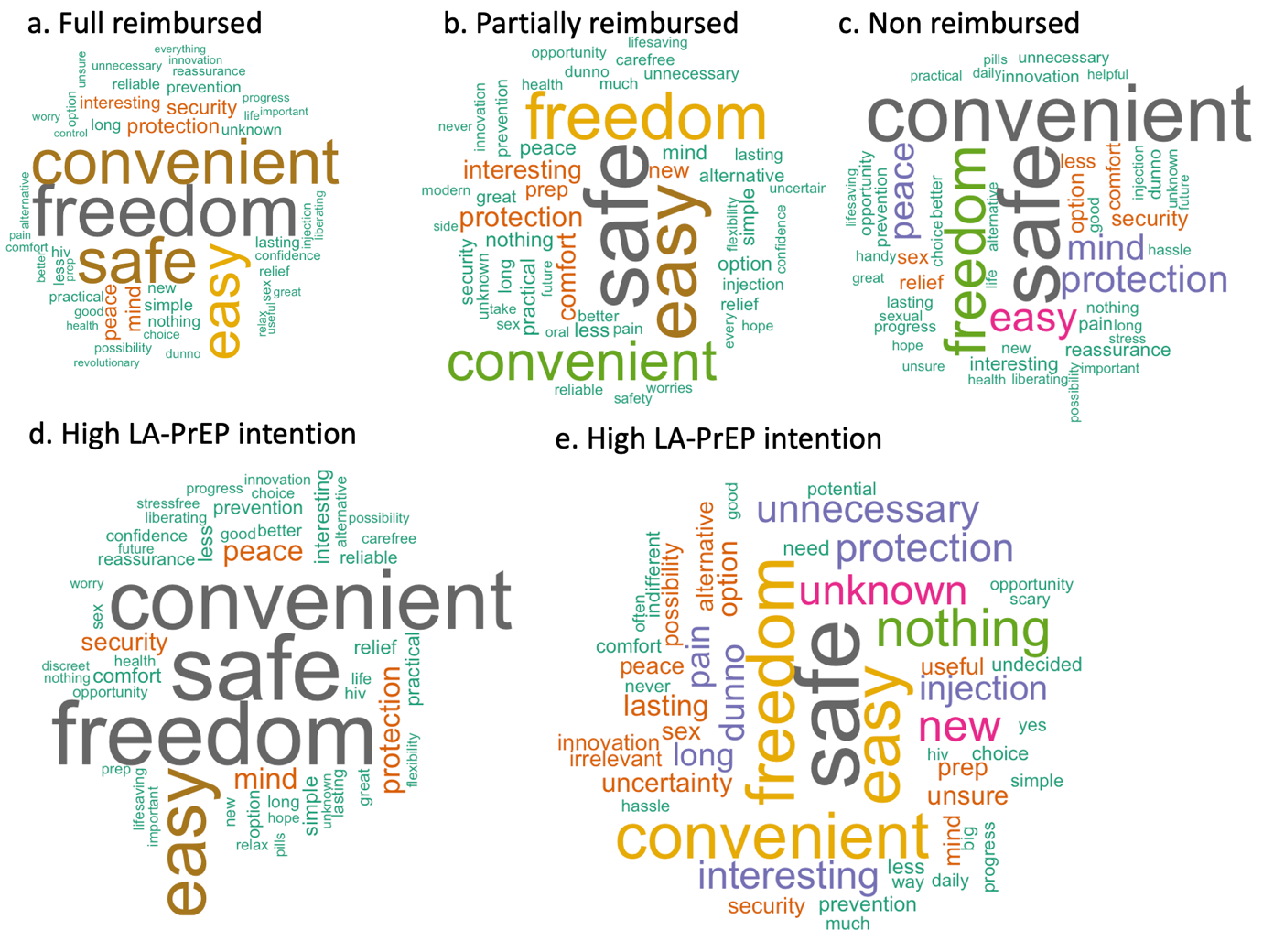


**Figure S5. Diagnostic values by number of topics in the structural topic modelling**

**
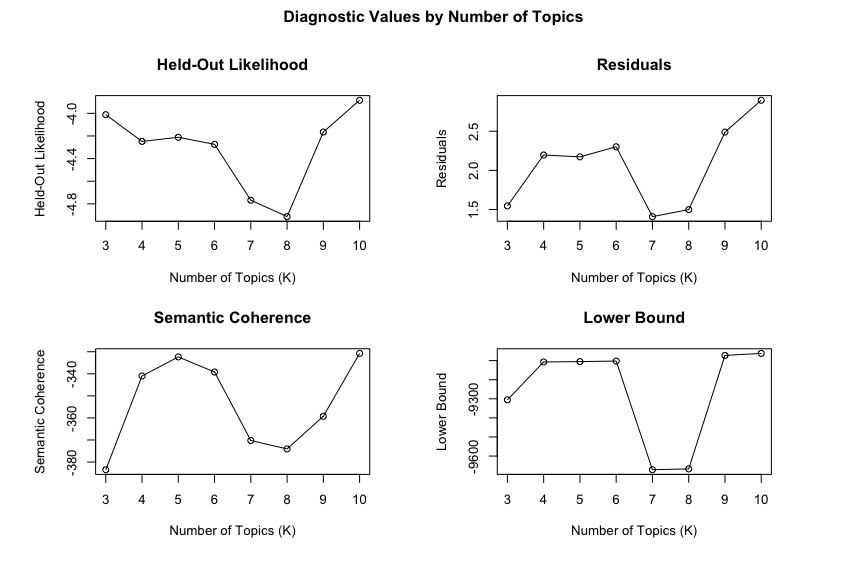
**

**Figure S6. Distribution of MAP estimates of Document-Topic Proportion in the structural topic modelling**

**
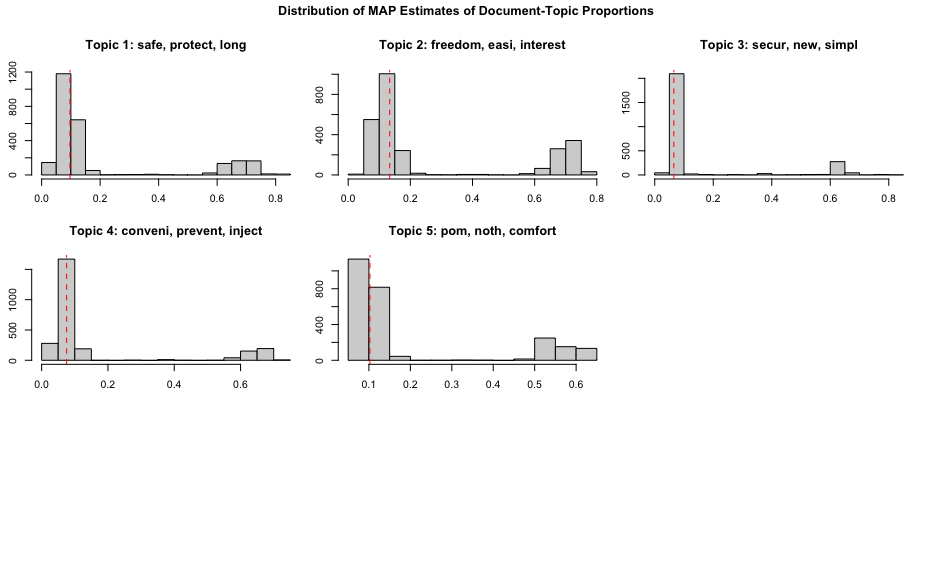
**

**Table S1 The country of residence among migrants**

| **Country** | **n** | **Percentage (%)** |
| --- | --- | --- |
| Austria | 33 | 2,0 |
| Belgium | 67 | 4,0 |
| Switzland | 44 | 2,7 |
| Cyprus | 15 | 0,9 |
| Czech Republic | 15 | 0,9 |
| Germany | 214 | 12,9 |
| Denmark | 45 | 2,7 |
| Spain | 107 | 6,5 |
| Finland | 33 | 2,0 |
| France | 64 | 3,9 |
| United Kingdoms | 476 | 28,7 |
| Greece | 11 | 0,7 |
| Ireland | 148 | 8,9 |
| Italy | 14 | 0,8 |
| Luxemburg | 20 | 1,2 |
| Netherlands | 194 | 11,7 |
| Noway | 40 | 2,4 |
| Other | 29 | 1,8 |
| Poland | 9 | 0,5 |
| Portugal | 41 | 2,5 |
| Sweden | 37 | 2,2 |

**Table S2. Sensitivity analysis of applying different word profiling methods**

| **Topic** | **Relevance** | **Word profiling methods** | **Words** |
| --- | --- | --- | --- |
| Topic 1 | 20.3% | Highest Prob | safe, protect, long, last, great, worry |
|  |  | FREX | long, safe, last, protect, access, worry |
|  |  | Lift | access, long, last, safe, worry, protect |
|  |  | Score | safe, protect, long, last, woryi, great |
| Topic 2 | 9.5% | Highest Prob | freedom, easy, interesting, alternative, pain, better |
|  |  | FREX | easy, interesting, freedom, better, pain, alternative |
|  |  | Lift | easy, interesting, better, freedom, pain, alternative |
|  |  | Score | freedom, easy, interest, altern, pain, better |
| Topic 3 | 14.7% | Highest Prob | secure, new, simple, practical, reassurance, prep |
|  |  | FREX | reassurance, secure, practical, simple, innovative, use |
|  |  | Lift | reassurance, use, innovation, practical, secure, opportunity |
|  |  | Score | secure, reassurance, new, simple, practical, use |
| Topic 4 | 4.9% | Highest Prob | convenient, prevent, inject, reliable, hope, hiv |
|  |  | FREX | convenient, prevent, reliable, hope, inject, hiv |
|  |  | Lift | convenient, reliable, hope, prevent, inject, hiv |
|  |  | Score | convenient, prevent, reliable, inject, hope, hiv |
| Topic 5 | 41.7% | Highest Prob | pom, noth, comfortable, option, NA, relief |
|  |  | FREX | nothing, option, unknown, NA, unnecessary, dunno |
|  |  | Lift | unknown, option, unnecessary, nothing, dunno, uncertainty |
|  |  | Score | pom, unknown, nothing, comfortable, NA |
