# Supplementary material for: Perceptions of Long-Acting Injectable Pre-Exposure Prophylaxis Among Men Who Have Sex With Men and Transgender Individuals in Europe Using Structural Text Modeling Technique: Cross-Sectional Study
Source: JMIR Public Health Surveill. 2025 Sep 12;11:e72491. doi: 10.2196/72491 (PMC12475884; doi:10.2196/72491)
Supplement: Multimedia Appendix 4 [file publichealth_v11i1e72491_app4.docx]

**Topic regression of the open responses of “What LA-PrEP means to you?” question from 3123 HIV-negative MSM and trans* individuals who completed the PROTECT survey in English across 20 European countries, October 2023-April 2024**

| Topic | Covariate | | B | 95% CI | *P* value |
| --- | --- | --- | --- | --- | --- |
| Topic 1: safety | Perceived high income | High | ref | - | - |
|  |  | Low | 0.023 | (–0.004 to 0.05) | .101 |
|  | Education | Bachelor or above | ref | - | - |
|  |  | Below a Bachelor’s degree | 0.052 | (0.022-0.083) | <.001 |
|  | Migration status | Nonmigrant | ref | - | - |
|  |  | Migrant | -0.011 | (–0.036 to 0.015) | .419 |
|  | Oral PrEP use | Noncurrent users | ref | - | - |
|  |  | Current users | -0.094 | (–0.123 to 0.066) | <.001 |
|  | Oral PrEP regimen * | Daily | ref | - | - |
|  |  | Event-driven | 0.030 | (–0.015 to 0.075) | .195 |
|  |  | Mixed use | -0.023 | (–0.074 to 0.029) | .389 |
|  | Oral PrEP adherence # | Optimal | ref | - | - |
|  |  | Suboptimal | 0.032 | (–0.014 to 0.077) | .174 |
|  | PrEP affordability | Fully reimbursed | ref | - | - |
|  |  | Partially reimbursed | 0.027 | (–0.004 to 0.057) | .085 |
|  |  | Nonreimbursed | 0.035 | (0.002-0.069) | .039 |
|  | Long-acting PrEP intention | No | ref | - | - |
|  |  | Yes | 0.019 | (–0.013 to 0.051) | .251 |
| Topic 2: empowerment | Perceived high income | High | ref | - | - |
|  |  | Low | -0.021 | (-0.049 to 0.006) | .131 |
|  | Education | Bachelor or above | ref | - | - |
|  |  | Below a Bachelor’s degree | -0.023 | (–0.06 to 0.015) | .237 |
|  | Migration status | Nonmigrant | ref | - | - |
|  |  | Migrant | 0.002 | (–0.026 to 0.03) | .892 |
|  | Oral PrEP use | Noncurrent users | ref | - | - |
|  |  | Current users | 0.070 | (0.042-0.097) | <.001 |
|  | Oral PrEP regimen * | Daily | ref | - | - |
|  |  | Event-driven | 0.005 | (–0.041 to 0.051) | .835 |
|  |  | Mixed use | 0.039 | (–0.031 to 0.109) | .274 |
|  | Oral PrEP adherence # | Optimal | ref | - | - |
|  |  | Suboptimal | 0.030 | (–0.017 to 0.077) | .218 |
|  | PrEP affordability | Fully reimbursed | ref | - | - |
|  |  | Partially reimbursed | -0.004 | (–0.035 to 0.027) | .795 |
|  |  | Nonreimbursed | -0.052 | (–0.087 to 0.017) | .004 |
|  | Long-acting PrEP intention | No | ref | - | - |
|  |  | Yes | 0.051 | (0.017-0.085) | .003 |
| Topic 3: peace of mind | Perceived high income | High | ref | - | - |
|  |  | Low | 0.001 | (–0.026 to 0.027) | .968 |
|  | Education | Bachelor or above | ref | - | - |
|  |  | Below Bachelor’s degree | -0.007 | (–0.037 to 0.023) | .638 |
|  | Migration status | Nonmigrant | ref | - | - |
|  |  | Migrant | 0.010 | (-0.014 to 0.033) | .422 |
|  | Oral PrEP use | Noncurrent users | ref | - | - |
|  |  | Current users | -0.001 | (–0.025 to 0.023) | .953 |
|  | Oral PrEP regimen* | Daily | ref | - | - |
|  |  | Event-driven | -0.011 | (–0.051 to 0.03) | .607 |
|  |  | Mixed use | 0.000 | (–0.053 to 0.054) | .989 |
|  | Oral PrEP adherence # | Optimal | ref | - | - |
|  |  | Suboptimal | -0.013 | (–0.058 to 0.031) | .559 |
|  | PrEP affordability | Fully reimbursed | ref | - | - |
|  |  | Partially reimbursed | -0.003 | (–0.035 to 0.03) | .88 |
|  |  | Nonreimbursed | -0.006 | (–0.037 to 0.026) | .722 |
|  | Long-acting PrEP intention | No | ref | - | - |
|  |  | Yes | -0.026 | (–0.055 to 0.002) | .067 |
| Topic 4Convenience and reliability | Perceived high income | High | ref | - | - |
|  |  | Low | -0.010 | (–0.035 to 0.015) | .436 |
|  | Education | Bachelor or above | ref | - | - |
|  |  | Below Bachelor’s degree | -0.029 | (–0.06 to 0.002) | .070 |
|  | Migration status | Nonmigrant | ref | - | - |
|  |  | Migrant | -0.007 | (–0.031-0.017) | .557 |
|  | Oral PrEP use | Noncurrent users | ref | - | - |
|  |  | Current users | 0.057 | (0.034-0.081) | <.001 |
|  | Oral PrEP regimen * | Daily | ref | - | - |
|  |  | Event-driven | -0.037 | (–0.078 to 0.003) | .072 |
|  |  | Mixed use | -0.031 | (–0.081 to 0.019) | .224 |
|  | Oral PrEP adherence # | Optimal | ref | - | - |
|  |  | Suboptimal | -0.026 | (–0.069 to 0.016) | .227 |
|  | PrEP affordability | Fully reimbursed | ref | - | - |
|  |  | Partially reimbursed | -0.034 | (–0.064 to 0.005) | .022 |
|  |  | Nonreimbursed | -0.002 | (–0.035 to 0.031) | .895 |
|  | Long-acting PrEP intention | No | ref | - | - |
|  |  | Yes | 0.032 | (0.003-0.062) | .033 |
| Topic 5 Concerns and uncertainties | Perceived high income | High | ref | - | - |
|  |  | Low | 0.008 | (–0.015 to 0.031) | .503 |
|  | Education | Bachelor or above | ref | - | - |
|  |  | Below Bachelor’s degree | 0.006 | (–0.023 to 0.035) | .676 |
|  | Migration status | Nonmigrant | ref | - | - |
|  |  | Migrant | 0.006 | (–0.017 to 0.03) | .588 |
|  | Oral PrEP use | Noncurrent users | ref | - | - |
|  |  | Current users | -0.032 | (–0.056 to 0.007) | .011 |
|  | Oral PrEP regimen^a^ | Daily | ref | - | - |
|  |  | Event-driven | 0.013 | (–0.023 to 0.048) | .483 |
|  |  | Mixed use | 0.014 | (–0.031 to 0.059) | .535 |
|  | Oral PrEP adherence^b^ | Optimal | ref | - | - |
|  |  | Suboptimal | -0.021 | (–0.058 to 0.015) | .255 |
|  | PrEP affordability | Fully reimbursed | ref | - | - |
|  |  | Partially reimbursed | 0.014 | (–0.013 to 0.041) | .318 |
|  |  | Nonreimbursed | 0.025 | (–0.007 to 0.056) | .132 |
|  | Long-acting PrEP intention | No | ref | - | - |
|  |  | Yes | –0.075 | (–0.101 to 0.05) | <.001 |

^a^available only to those who reported currently using oral PrEP.

^b^available only to those who reported currently using daily or event-driven PrEP.
